# Supplementary material for: Empirical methods for controlling false positives and estimating confidence in ChIP-Seq peaks
Source: BMC Bioinformatics. 2008 Dec 5;9:523. doi: 10.1186/1471-2105-9-523 (PMC2628906; doi:10.1186/1471-2105-9-523)
Supplement: Additional File 1 — A variety of html documents from the USeq web site detailing the available applications, their best usage, output file type descriptions, command line menus, etc. [file 1471-2105-9-523-S1.zip › USeqUserGuides/JavaDoc.zip!/JavaDoc/trans/cel/CelProcessor.html]

CelProcessor


|  |  |  |
| --- | --- | --- |
| **Overview** \| **Package** \| **Class** \| **Use** \| **Tree** \| **Deprecated** \| **Index** \| **Help** | |  |
| **PREV CLASS**   **NEXT CLASS** | **FRAMES**    **NO FRAMES**     **All Classes** |  |
| SUMMARY: NESTED \| FIELD \| CONSTR \| METHOD | DETAIL: FIELD \| CONSTR \| METHOD |  |


---


## trans.cel Class CelProcessor

```
java.lang.Object
  trans.cel.CelProcessor
```

---

``` public class CelProcessor extends java.lang.Object ```

Application to process '.cela' files, wrapper for `CelMapper`,
`QuantileNormalization`.

---

**Constructor Summary**

`CelProcessor(java.lang.String[] args)`


| **Method Summary** | |
| --- | --- |
| `static boolean` | `anyNaNs(float[] ints)`             Looks for NaN in IntensityFeatures. |
| `static boolean` | `anyNaNs(IntensityFeature[] ints)`             Looks for NaN in IntensityFeatures. |
| `static void` | `breakAndSaveCelp(java.io.File celpFile, MapFeature[] features)`             Given a float[] celp file and the corresponding MapFeature[] will make a minimal |
| `static void` | `breakSaveFeatureArray(java.util.ArrayList bpmapInfo, MapFeature[] mapFeatures, java.io.File celpFile)`             Use this method to break and save a celp float[] file into chromosomal components in a directory named after the celpFile minus the .celp extension. |
| `static void` | `main(java.lang.String[] args)` |
| `static void` | `printDocs()` |
| `void` | `processArgs(java.lang.String[] args)`             This method will process each argument and assign any new varibles |

**Methods inherited from class java.lang.Object**

`equals, getClass, hashCode, notify, notifyAll, toString, wait, wait, wait`

**Constructor Detail**

### CelProcessor

```
public CelProcessor(java.lang.String[] args)
```


**Method Detail**

### breakSaveFeatureArray

```
public static void breakSaveFeatureArray(java.util.ArrayList bpmapInfo,
                                         MapFeature[] mapFeatures,
                                         java.io.File celpFile)
```

:   Use this method to break and save a celp float[] file into chromosomal components in a
    directory named after the celpFile minus the .celp extension.

---


### anyNaNs

```
public static boolean anyNaNs(IntensityFeature[] ints)
```

:   Looks for NaN in IntensityFeatures. Returns true if NaNs found, false if clean.

---


### anyNaNs

```
public static boolean anyNaNs(float[] ints)
```

:   Looks for NaN in IntensityFeatures. Returns true if NaNs found, false if clean.

---


### breakAndSaveCelp

```
public static void breakAndSaveCelp(java.io.File celpFile,
                                    MapFeature[] features)
```

:   Given a float[] celp file and the corresponding MapFeature[] will make a
    minimal

---


### processArgs

```
public void processArgs(java.lang.String[] args)
```

:   This method will process each argument and assign any new varibles

---


### printDocs

```
public static void printDocs()
```

---


### main

```
public static void main(java.lang.String[] args)
```


---


|  |  |  |
| --- | --- | --- |
| **Overview** \| **Package** \| **Class** \| **Use** \| **Tree** \| **Deprecated** \| **Index** \| **Help** | |  |
| **PREV CLASS**   **NEXT CLASS** | **FRAMES**    **NO FRAMES**     **All Classes** |  |
| SUMMARY: NESTED \| FIELD \| CONSTR \| METHOD | DETAIL: FIELD \| CONSTR \| METHOD |  |


---
